# Carbon Dot-Laponite Hybrid Nanocomposites as Selective Turn-Off Sensors for Hg^2+^ Detection and Photoluminescence Quenching Mechanism

**DOI:** 10.1021/acsomega.4c07183

**Published:** 2024-12-17

**Authors:** Bruno S. D. Onishi, Albano N. Carneiro Neto, Sidney J. L. Ribeiro

**Affiliations:** † Institute of Chemistry, São Paulo State University—UNESP, São Paulo, Araraquara 14800-060, Brazil; ‡ Physics Department and CICECO − Aveiro Institute of Materials, University of Aveiro, 3810-193 Aveiro, Portugal

## Abstract

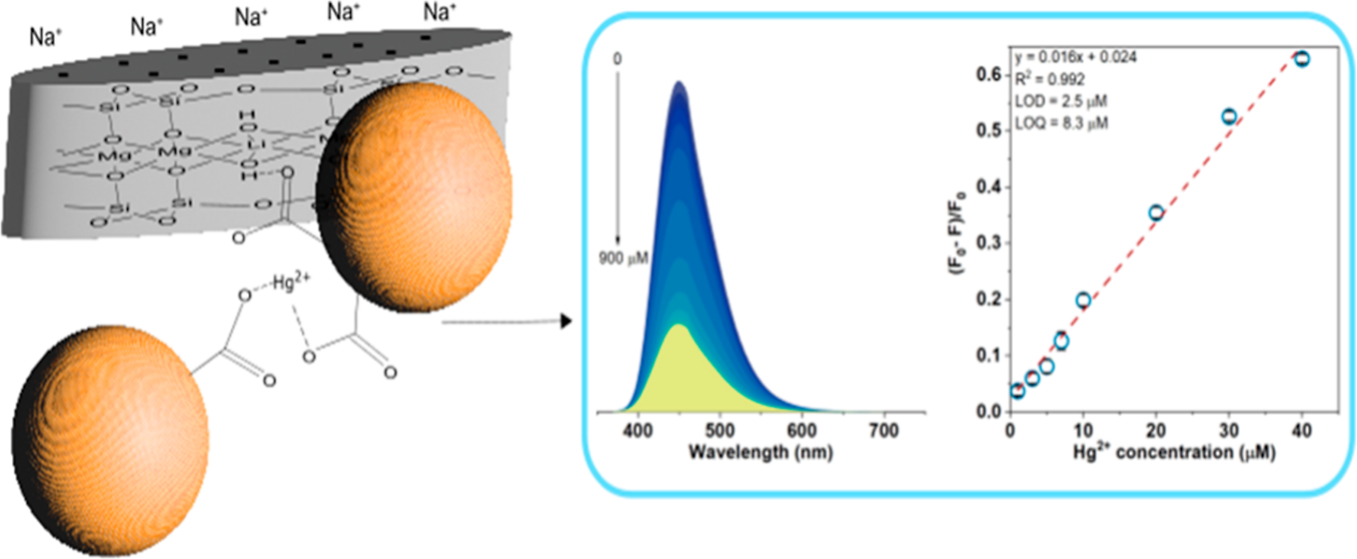

Motivated by the importance of Hg^2+^ detection in water due to its harmful effect on the environment and human health, we investigated a recently developed nanocomposite based on carbon dots (CDs) and LAPONITE as an optical chemical sensor using photoluminescence emission. While several studies have reported the Hg^2+^ detection using CDs’ photoluminescence emission, there is a lack of in-depth investigation into the quenching mechanisms involved in turn-off sensors. In this study, we propose a Stern–Volmer analysis at three different temperatures (288, 298, and 303 K). The results indicated selectivity for Hg^2+^ over that of the other evaluated metal. The optimum detection range for Hg^2+^ was found to be 1–40 μM, with limits of detection and quantification of 2.5 and 8.3 μM, respectively. Using the Stern–Volmer models, we found that static quenching dominates over collisional quenching, possibly due to the complexation between nanocomposite’s carboxylate groups and Hg^2+^. Additionally, the modified Stern–Volmer model, which accounts for the fractional accessibility of the fluorophores by the quenchers, suggests that some parts of the sensor are inaccessible to the quencher.

## 1 Introduction

Due to the harmful effects of heavy metals on both human health and the environment, monitoring strategies in water bodies are essential for detection and quantification. Metal ions such as Cd^2+^, Cr^6+^, Co^2+^, Cu^2+^, Pb^2+^, Hg^2+^, Zn^2+^, As^3+^, and Ag^+^, even in trace amounts, can pose significant risks to humans, flora, and fauna. Studies have linked elevated concentrations of these contaminants to industrial and urban waste, stemming from inadequate waste treatment practices and industrial mishaps.^1−3^ The toxicity of these metals often arises from their binding to nonessential sites intended for essential metals in physiological processes. Among these, Hg^2+^ is notably hazardous due to its strong affinity for thiol groups, forming covalent bonds with cysteine residues in proteins. This interaction triggers the production of reactive oxygen species, lowers antioxidant levels, and promotes the accumulation of free radicals, which can damage cellular DNA and potentially initiate carcinogenic processes.^4−6^

Analytical instruments such as atomic absorption spectroscopy, inductively coupled plasma mass spectrometry, X-ray fluorescence spectroscopy, and voltammetry are commonly employed for quantifying heavy metals.^7^ However, these methods often suffer from drawbacks, including high costs, lengthy analysis times, complex instrumentation, and the requirement for extensive sample preparation. In contrast, optical chemical sensors offer a cost-effective alternative. These sensors utilize the interaction of electromagnetic field with the analyte to produce an analytical signal based on optical parameters such as absorption, emission, lifetime, and anisotropy, which can directly correlate with concentration.^8,9^

Photoluminescence (PL) emission intensity serves as a pivotal measurement in optical sensing. When an analyte induces quenching, a direct relationship can be established between the quencher’s concentration and the reduction in emission intensity. This relationship is typically quantified by constructing an analytical curve, enabling precise detection and quantification of the quencher analyte’s concentration. This fundamental principle underpins the operation of photoluminescent chemical sensors.^10,11^

Due to their straightforward synthetic routes and optical properties, such as tunable PL and high quantum yield (QY), carbon dots (CDs) find versatile applications in photonics.^12−14^ They are widely recognized for their utility as sensors, serving both as chemical sensors for detecting and quantifying ions and molecules, and as physical sensors like temperature sensors.^15−17^ Their solubility in aqueous media and easily modifiable surfaces make CDs promising candidates for diverse applications in biological and environmental settings, enhancing selectivity toward specific analytes. Moreover, their large surface area and sensitive surface states facilitate interactions with the environment, enabling efficient detection through mechanisms such as fluorescence quenching, which is an advantageous characteristic in PL chemical sensors.

Recently, the solid-state emission from a nanocomposite based on carbon dots embedded in LAPONITE was the focus of study by our group.^18^ However, we observed significant emission not only in the solid state but also in aqueous suspension with a high absolute quantum yield (QY = 0.22). Our analysis highlighted the prevalence of carboxylate groups within the nanocomposite structure, attributable to LAPONITE incorporation, which enhances potential interactions with Hg^2+^ ions. This discovery prompted further investigation of its viability as a PL chemical sensor for Hg^2+^. Additionally, a problem arises with metal selectivity, where interference from metal ions other than Hg^2+^, such as Fe^2+^, Fe^3+^, Co^2+^, Ni^2+^, Cd^2+^, and Pb^2+^, may occur.^19,20^ Among the nanocomposites based on LAPONITE-carbon dot or other clay minerals (e.g., bentonite and halloysite), to the best of our knowledge, we found only one report paper involving the application as a PL chemical sensor, which used halloysite-carbon dot for Fe^3+^ sensing,^21^ but the authors did not include Hg^2+^ in the evaluated metals. The works reported in the literature aimed applications such as white light emission diodes (WLEDs),^22^ multilayer films,^23^ active food packings,^24^ multilevel logic digital encryption and sensing perfluorinated pollutants,^25^ photocatalysis,^26^ and adsorbents.^27^

While numerous studies exist on Hg^2+^ sensing, few delve deeply into the quenching mechanism, typically involving electron transfer from excited states of carbon dots to Hg^2+^, as inferred from emission decay measurements.^19,20,28−33^ Our study proposes investigating this phenomenon across three different temperatures (288, 298, and 303 K), employing Stern–Volmer and modified Stern–Volmer models. These models will provide insights into the tendencies of the PL quenching mechanism under varying conditions.

## 2 Experimental Section

### 2.1 Materials

The synthesis and characterization of the carbon dot-LAPONITE nanocomposite (CDLP-D) and the CD (carbon dot sample without LAPONITE) are detailed in previous work.^18^ This material revealed that the carbon dots, with an average size of 2.8 ± 0.5 nm, as determined by STEM, interact with the nanodisc-shaped LAPONITE (25 nm diameter) via carboxylate groups on the carbon dots, which preferentially associate with the Mg octahedra at the edges of the LAPONITE structure.

The samples used were CDLP-D (carbon dot-LAPONITE nanocomposite) and CD (sample without LAPONITE). For the metal sensing assay, the following salts were used: MnCl_2_·4H_2_O (Acros), FeCl_3_·6H_2_O (Synth), FeCl_2_·4H_2_O (Merck), CoCl_2_ (Synth), NiCl_2_·6H_2_O (Synth), CdCl_2_·H_2_O (Êxodo Cientifica), PbCl_2_ (Neon), Zn(NO_3_)_2_ (Synth), CuSO_4_ (LAFAN), AgNO_3_ (Sigma), and HgCl_2_ (Reagen). For the anions, the salts used were NaCl (Neon), NaNO_3_ (Synth), Na_2_CO_3_ (Synth), Zn(SO_4_)_2_ (Vetec), Na_3_PO_4_, and NaAsO_2_ (Reagen). The KCl for ionic strength assay was purchased from Synth and HCl and NaOH for pH adjustment from Sigma-Aldrich.

### 2.2 Photostability Measurements

The PL measurements were also carried out on a Horiba-Jobin Yvon Fluorolog 3 FL3-22 spectrofluorometer equipped with a Hamamatsu R928P photomultiplier. A continuous xenon lamp FL-1039A/40A 450 W was used for the excitation and emission spectra. For the PL decay measurement, a pulsed diode light source NanoLED-350 (Horiba-Jobin Yvon) was used with a wavelength of 350 nm.

For the pH experiments, suspensions of 0.1 mg·mL^–1^ CDLP-D or CD in ultrapure water (Milli-Q) were prepared, and the pH was adjusted using NaOH or HCl as needed. Each pH condition, ranging from 2 to 11, was tested in duplicate. PL emission spectra were recorded for each pH-adjusted sample, and Figure S1a displays the averaged spectra for each pH condition.

For the sensor concentration assay, the CDLP-D 5.0 mg·mL^–1^ water suspension was diluted for concentrations of 0.01, 0.05, 0.1, 0.3, 0.5, 1.0, 2.0, 3.0, and 4.0 mg·mL^–1^. The PL emission was measured for each sample, including 5.0 mg·mL^–1^. The same procedure was applied for CD but in the range of 0.01–0.5 mg·mL^–1^ using a suspension of 1.0 mg·mL^–1^.

The photostability as a function of time was assessed at a controlled temperature of 298 K using an FL-1027 single-position thermostatic cell holder, which was coupled with an external circulating temperature bath pumping a mixture of ethylene glycol and water. The holder also included a magnetic stirrer to agitate the suspension during the measurements. This system was integrated with the spectrofluorometer. Spectra were collected every hour for 8 h, during which the CDLP-D or CD 0.1 mg·mL^–1^ water suspension remained continuously irradiated by the xenon lamp at 360 nm.

The ionic strength assay was conducted by suspending 0.1 mg·mL^–1^ of CDLP-D or CD samples in KCl solutions, with concentrations ranging from 0.1, 0.3, 0.5, 0.7, and 0.9 M, prepared by diluting a 1.0 M KCl solution. This assay was performed in duplicate, and the PL emission was measured for each sample.

### 2.3 Sensing and Stern–Volmer Assay

Except for Zn^2+^, Ag^+^, and Cu^2+^, prepared from Zn(NO_3_)_2_·6H_2_O, Ag(NO_3_), and CuSO_4_, all metals were prepared from chloride salts. For the anions, sodium salt was used, except for SO_4_^2–^, which was used in Zn(SO_4_)_2_. The primary solution was prepared with a concentration of 1000 μM in ultrapure water and diluted to 100 μM, into which the 0.1 mg·mL^–1^ CDLP-D or CD samples were suspended. The assay was conducted in triplicate with a volume of 1.0 mL, and PL emissions were measured for each sample. Before the measurements, the samples were incubated at 295 K for 10 min. The same procedure was applied for Hg^2+^ and Fe^3+^, but the CDLP-D or CD was suspended in a broader concentration range of 0–900 μM.

The Stern–Volmer assay was made using 0.1 mg·mL^–1^ CDLP-D with Hg^2+^ solution at a concentration of 0–20 μM similarly to the sensing assay. However, in this case, the temperature remained constant at 288, 298, or 313 K using the FL-1027 single-position thermostatic cell holder. The choice of these temperatures aligns with standard conditions in quenching studies to distinguish between collisional and static quenching, as outlined in the literature.^34−39^ In addition, this temperature range reflects typical environmental conditions, covering cooler to warmer scenarios that may be encountered in real-world monitoring applications.

## 3 Results and Discussion

### 3.1 Photostability

**Figure 1:**
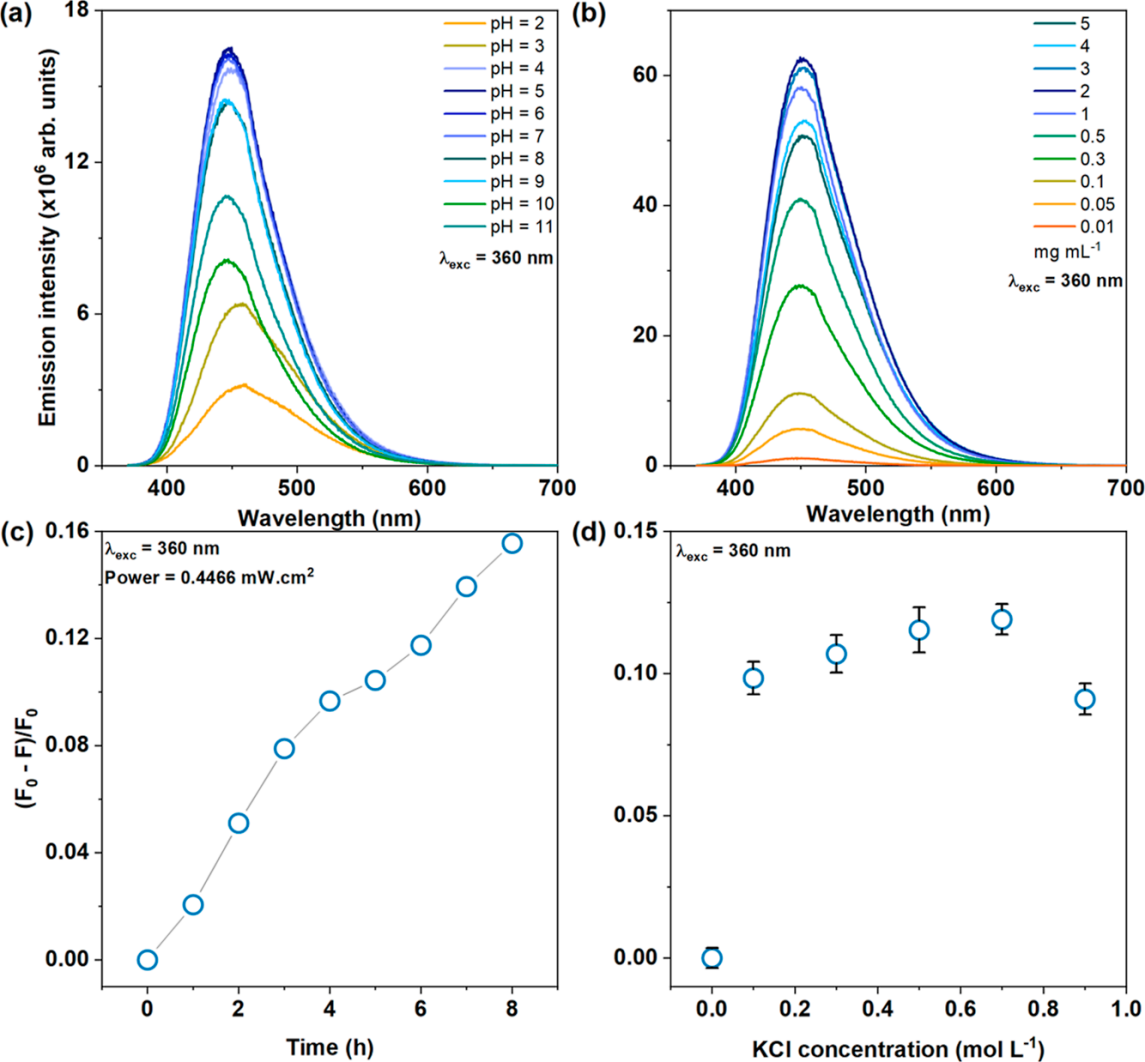
Emission spectra of CDLP-D as a function of (a) pH and (b) concentration; relative integrated area of emission band as a function of (c) time and (d) ionic strength—*F*_0_ is the integrated area of the CDLP emission spectrum at (c) 0 h and (d) 0 mol·L^–1^ KCl, and *F* is the integrated area of the CDLP emission spectrum at (c) *x* (*x* = 1 to 8) hours and (d) *y* (*y* = 0.1 to 0.9) mol·L^–1^. For (a,c,d), the CDLP concentration was maintained at 0.1 mg·mL^–1^.

According to the PL emission spectra presented in Figure 1a, the emission intensity of CDLP-D remains stable in the range of pH = 4–7, with minor quenching in pH 8 and 9 and considerable quenching in extreme pH conditions (pH = 2 and 3; 10 and 11). In addition, in acid pH (2 and 3), it is possible to notice the red shift in emission spectra. Liu et al. demonstrated in their review three possible mechanisms for the pH-dependent PL mechanisms for CDs: (1) change of energy level, (2) formation of aggregation-induced state, and (3) pH-induced electron transfer.^40^ Based on previous results,^18^ the surface of CDLP-D is predominantly composed of carboxyl and hydroxyl groups, which can protonate and deprotonate in acidic and basic pH conditions, respectively, potentially affecting the energy level state.^18,36−38^ In acidic pH, the higher concentration of H^+^ can also contribute to aggregation-induced quenching.^44,45^ Conversely, the PL emission of the CDs without LAPONITE (Figure S1a) is less intense at basic pH levels (10 and 11) compared to CDLP-D. However, in the pH range of 4–8, there is no observable emission quenching. These results support the hypothesis that the emission of CDs originates from surface states due to functional organic groups. Nevertheless, the pH-dependent PL mechanisms in our samples are not entirely clear, and further studies examining PL decay, zeta potential, and UV–vis absorption as functions of pH are necessary, which are beyond the scope of this work.^41−43^

The PL emission intensity of CDLP-D increased from 0.01 to 2 mg·mL^–1^, followed by quenching as the concentration increased from 3 to 5 mg·mL^–1^, as shown in the PL emission spectra in Figure 1b. This quenching is likely due to the formation of an aggregation-induced state, which may quench the emission of CD samples.^46,47^ A similar effect occurs in the pure CD sample (Figure S1b), but in this case, quenching starts at 0.5 mg·mL^–1^, a lower concentration compared to CDLP-D.

The CDLP-D sample exhibited a 16% relative integrated area emission reduction after 8 h of continuous UV irradiation (Figure 1c), whereas the CD sample showed a 12% reduction over the same period (Figure S1c). Additionally, the CD sample demonstrated a higher photostability in terms of ionic strength (Figure S1d), with a reduction of less than 5% in the relative integrated emission, compared to the CDLP-D sample (Figure 1d), which had a reduction of less than 12%.

From an experimental point of view, the working concentration of CDLP-D for sensing assays was 0.1 mg·mL^–1^, with the suspension’s pH itself, which was 6.0. Each measurement took around 3 min of continuous UV light irradiation per sample. Given that only 2% of the integrated area was reduced after 1 h of continuous irradiation, the sample can be considered photostable within the measured time frame, equating to a proportional reduction of 0.1%. Despite the interference from a high concentration of KCl resulting in a 10% hindrance in PL emission, the concentration of the metal salts used fell within the μM range.

### 3.2 Selectivity and Sensitivity

**Figure 2:**
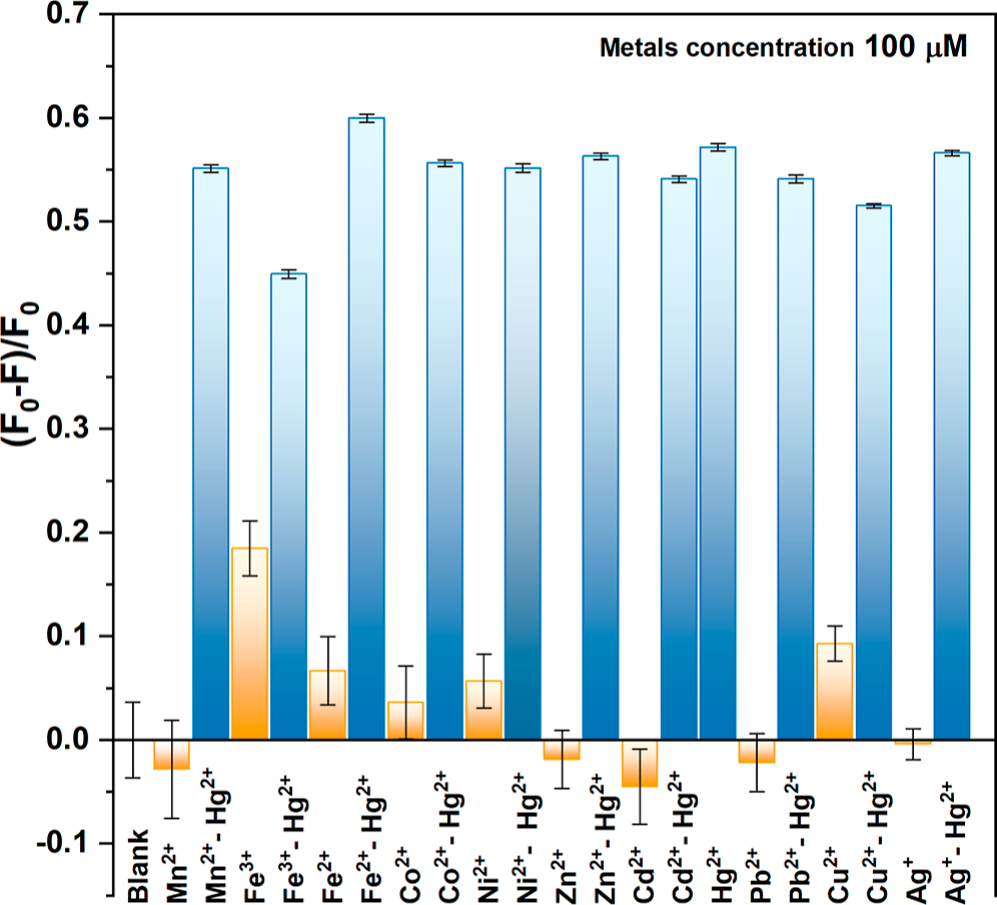
Relative emission integrated area of CDLP-D using 100 μM of Mn^2+^, Fe^3+^, Fe^2+^, Co^2+^, Ni^2+^, Zn^2+^, Cd^2+^, Pb^2+^, Cu^2+^, and Ag^+^ (yellow bars) and each one together with Hg^2+^ (blue bars). *F*_0_ is the blank integrated area emission (CDLP-D) and *F* is the integrated area emission in the presence of the respective metals.

**Figure 3:**
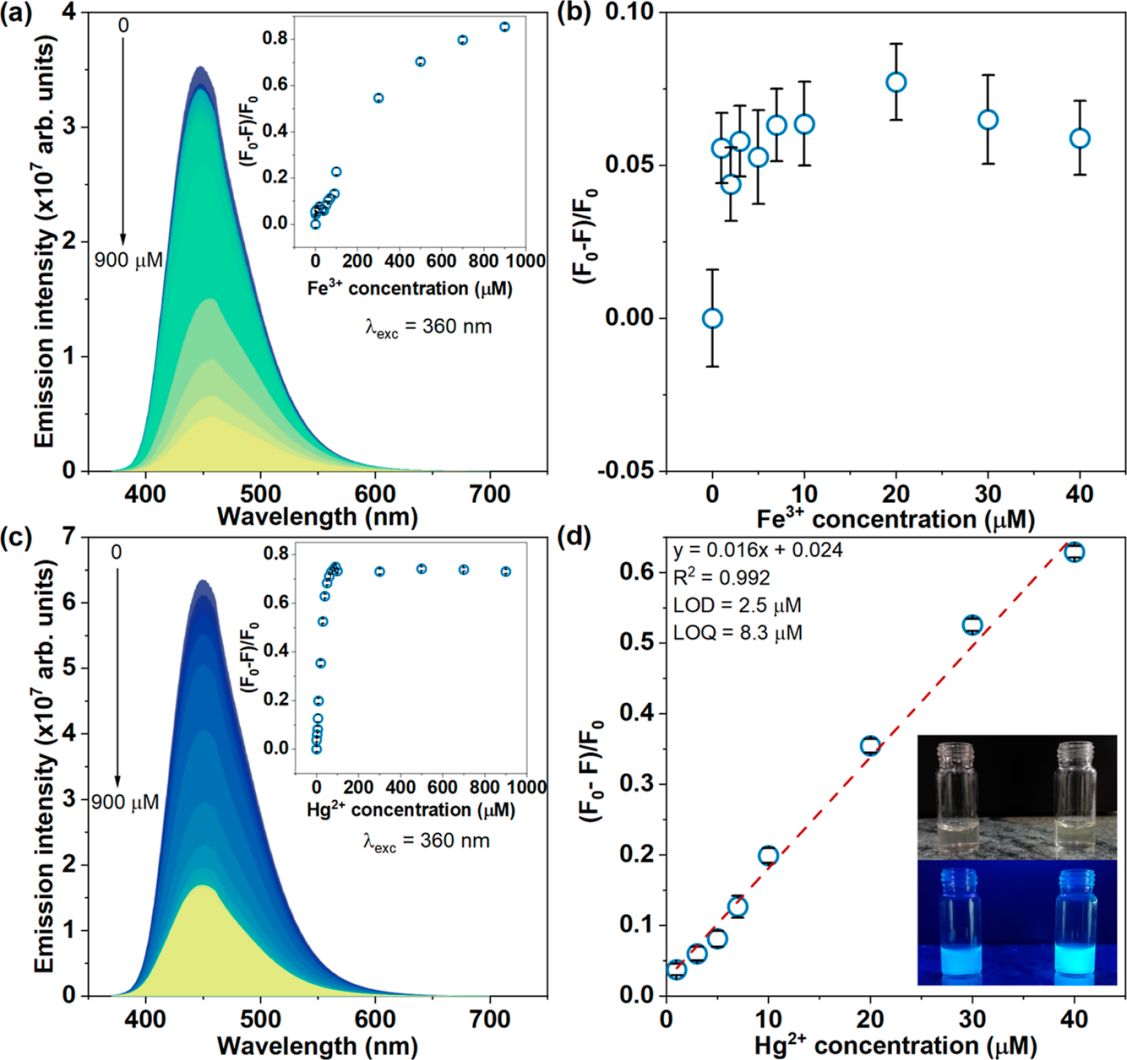
PL emission spectra of CDLP-D as a function of Fe^3+^ (a) and Hg^2+^ (b) concentrations from 0 to 900 μM. The inset graph in (a,c) is the relative integrated emission area as a function of Fe^3+^ and Hg^2+^ concentrations in the 0–900 μM range, respectively, as well as (b,d) in 1–40 μM. The inset picture in (d) represents the CDLP-D suspension with Hg^2+^ 50 μM (left) and without Hg^2+^ (right), where below it is possible to notice a less intense bright emission of CDLP-D in the presence of Hg^2+^, under 375 nm UV lamp irradiation.

To verify the selectivity of CDLP-D for Hg^2+^, PL sensing assays with other metal ions at 100 μM concentration such as Mn^2+^, Fe^2+^, Fe^3+^, Co^2+^, Ni^2+^, Zn^2+^, Cd^2+^, Pb^2+^, Cu^2+^, and Ag^+^ were performed, as shown in Figure 2. Notably, at a concentration of 100 μM Hg^2+^, there was a 58% reduction in the relative integrated area emission of CDLP-D, indicating significant quenching due to the interaction with Hg^2+^. In comparison, the integrated area was affected by less than 9% for other metals, except for Fe^3+^, which caused an 18% reduction. This could be due to the absorption of the same excitation wavelength of CDLP-D (λ_exc_ = 360 nm) by Fe^3+^ complex ions formed in water, such as Fe(OH)^2+^, at the working pH.^48^ However, in the sensor’s range of 1–40 μM, the integrated area was affected by less than 7%, as shown in Figure 3b. Additionally, other metals did not significantly affect the Hg^2+^ quenching, as shown by the blue bars in Figure 2. Anions such as Cl^–^, NO_3_^–^, CO_3_^2–^, SO_4_^2–^, PO_4_^3–^, and AsO_2_^–^ were evaluated at the same metal concentration, as shown in Figure S2. Among these, CO_3_^2–^ was the only anion that caused quenching, reducing the integrated emission area by 13%. While this effect is observed, it is important to note that these test conditions involve a much higher concentration than the typical working range of the nanocomposite. Additionally, arsenic, which commonly coexists with mercury in mining environments, does not exhibit significant quenching. This result supports the sensor’s applicability in real-world conditions.

The CDLP-D PL emission with an Fe^3+^ concentration was evaluated to verify the interference range in the measurements. Figure 3a depicts the emission spectra of CDLP-D in the 0–900 μM range of the Fe^3+^ concentration. Variation in emission spectra is noticeable only at high concentrations of Fe^3+^ (depicted as green to yellow) within the 100–900 μM range. However, in the 1–40 μM range, which is optimal for Hg^2+^ detection, the relative integrated emission area remained below 7%, as seen in Figure 3b, indicating minimal Fe^3+^ interference in this range.

For Hg^2+^, a plateau of quenching is observable in the 100–900 μM range (inset in Figure 3c), with the same PL emission variation (depicted as green-yellow). Conversely, in the 1–90 μM range, the emission spectra decrease in intensity as the Hg^2+^ concentration increases, depicted as blue to green-yellow. The optimum range for Hg^2+^ detection was found to be 1–40 μM, where a good linear calibration curve with a high coefficient of determination (*R*^2^ = 0.992) and a linear equation of *y* = 0.016*x* + 0.024 was established (Figure 3d). This equation can be used to quantify the Hg^2+^ concentration, where *y* = (*F*_0_ – *F*)/*F*_0_ and *x* = [Hg^2+^]. The limit of detection (LOD) and limit of quantification (LOQ) found by measuring 10 blank samples were 2.5 and 8.3 μM, respectively.^49^ These values are comparable to those found in previous works,^29−31^ although several reports have found lower LODs in the nM range,^32,33,50,51^ as can be seen in Table S1. The CDLP-D sensor, apart from the good selectivity for Hg^2+^, achieved a LOD of 2.5 μM within a linear range of 1–40 μM. These values demonstrate competitive sensitivity compared to other methods, underscoring the effectiveness of CDLP-D in detecting Hg^2+^.

For the pure CD sample, the optimum range for Hg^2+^ sensing was between 10 and 60 μM, with *R*^2^ = 0.986, a linear equation of *y* = 0.0058*x* + 0.10, and LOD and LOQ of 11.9 and 39.3 μM, respectively (Figure S3d). However, in this range, for the CD sample, the relative integrated emission area remained below 20% for Fe^3+^ ions (Figure S3b), indicating high interference of Fe^3+^ ions in Hg^2+^ detection, because the quenching remained below 40% of total emission in the Hg^2+^ sensing assay. These results indicate superior performance of the CDLP-D composite for Hg^2+^ detection compared with the pure CD sample.

### 3.3 Quenching Mechanism

To establish a possible PL emission quenching mechanism by Hg^2+^, two models were applied at three different temperatures (288, 298, and 313 K). Analysis at different temperatures could provide information about whether the mechanism is collisional or static quenching.^52^ The first model is described by Stern–Volmer, eq 1, where *K*_sv_ is the Stern–Volmer quenching constant, indicating the sensitivity of the fluorophore to a quencher.
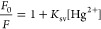
1

**Figure 4:**
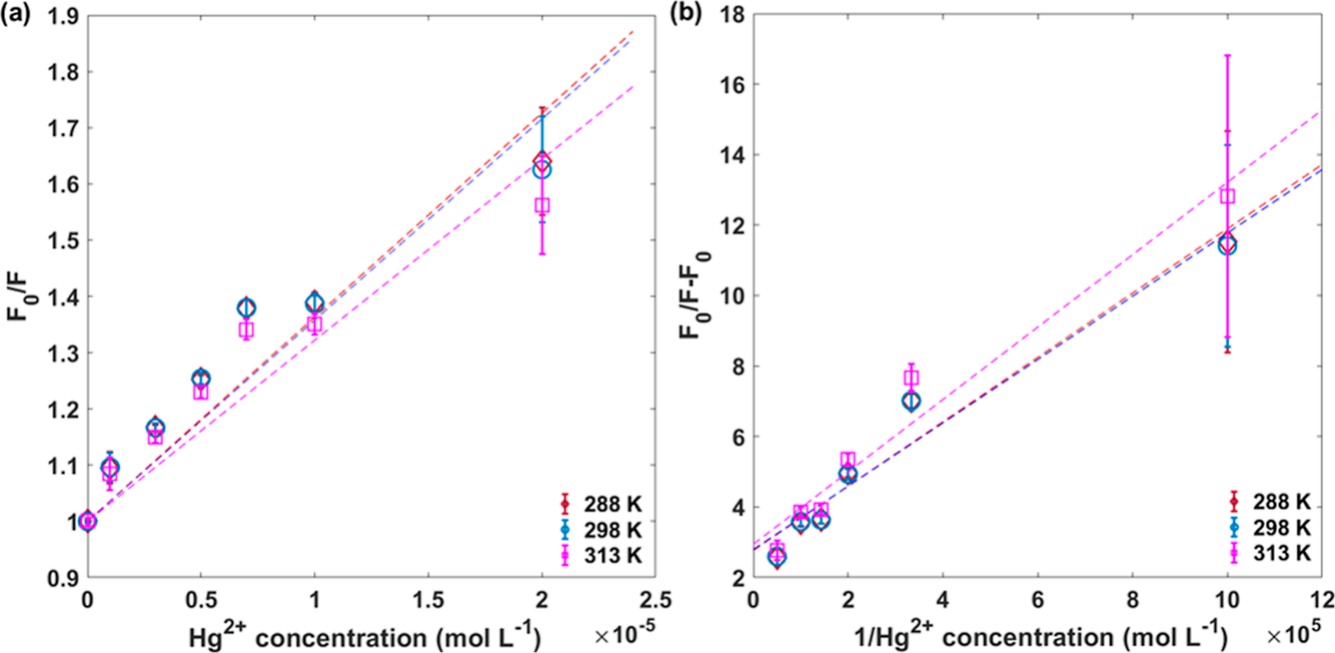
(a) Stern–Volmer and (b) modified Stern–Volmer plot at 288, 298, and 313 K.

As shown in Figure 4a, deviations from linearity are observed at all temperatures when applying the first model. This deviation can be associated with a high quencher concentration or, when the deviation is toward the *x*-axis, the presence of more than one fluorophore population.^52,53^ As mentioned in previous work,^18^ the CDLP-D emission originated from byproducts on the surface or the nanoparticle’s core (high energy) and surface states due to organic functional groups (low energy). Thus, this deviation is expected. The temperature enhancement resulted in lower *K*_sv_ values, presented in Table 1, which is not characteristic of collisional quenching behavior, since higher temperatures promote faster diffusion.^35,52^ Conversely, it is consistent with a static behavior with the formation of a nonfluorescent complex, in which the higher temperature promotes the dissociation of the quencher-fluorophore bond.^28,30^

**Table 1:** Stern–Volmer Quenching Constant (*K*_sv_) Obtained from the Slope of the Stern–Volmer Plot and the Stern–Volmer Quenching Constant Accessible Fraction (*K*_a_) Obtained from the Slope of the Modified Stern–Volmer Plot

	Stern–Volmer (eq 1)		modified Stern–Volmer (eq 2)		
temp. (K)	*K*_sv_ (M^–1^)	*R*^2^	*K*_a_ (M^–1^)	*f*_a_	*R*^2^
313	3.22 × 10^4^	0.869	2.87 × 10^5^	0.36	0.957
298	3.58 × 10^4^	0.855	3.11 × 10^5^	0.36	0.955
288	3.63 × 10^4^	0.855	3.05 × 10^5^	0.34	0.961

**Figure 5:**
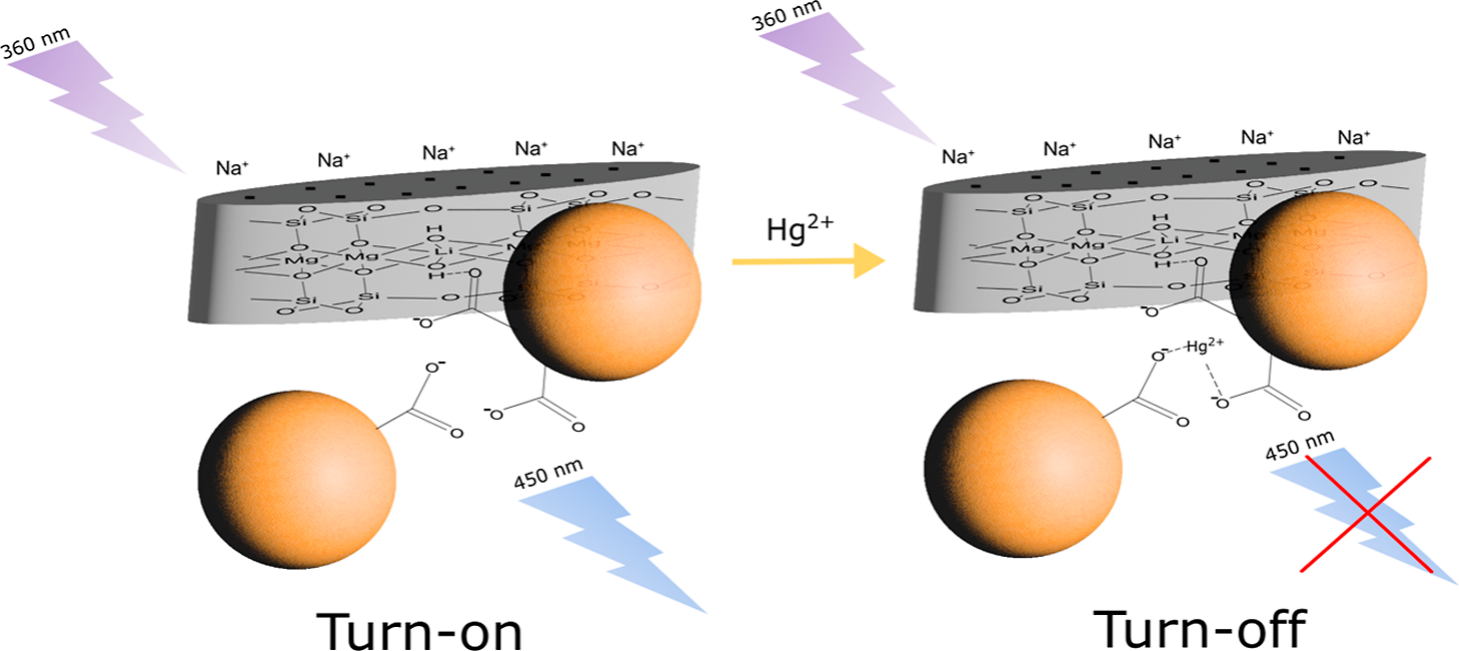
Scheme of the possible mechanism for Hg^2+^ sensing by CDLP-D.

In the case of collisional quenching, the quencher affects the excited state of the fluorophore by collisional interaction, with possible energy or charge transfer mechanisms involved.^10,27,29^ The results from the emission curve decay (Figure S5) revealed that at high Hg^2+^ concentrations (100 μM), the excited state was affected. The lifetime without Hg^2+^ was τ_0_ = 12.74 ± 0.03 ns, which decreased to τ_(100 μM)_ = 9.16 ± 0.03 ns with 100 μM (Figure S4). However, at low Hg^2+^ concentrations (ca. 10 μM), τ_(10 μM)_ = 12.05 ± 0.03 ns, indicating a static quenching in low concentrations of Hg^2+^.^54^ Thus, collisional quenching could occur at high Hg^2+^ concentrations. In addition, if we consider that the process is diffusion-limited, *K*_sv_ can be described as *K*_sv_ = *k*_q_τ_0_, where *k*_q_ is the bimolecular quenching constant, which in this case *k*_q_ = 2.81 × 10^12^ M^–1^·s^–1^. According to Lakowicz, a diffusion-controlled quenching is around *k*_q_ = 1 × 10^10^ M^–1^·s^–1^ and larger values indicate a binding interaction.^52^ In agreement with the results, the static quenching process appears to be dominant at low Hg^2+^ concentrations rather than the collisional process, but the latter cannot be disregarded, which is consistent with Stern–Volmer assays for similar systems in the literature.^29,30,55^ This could explain the selectivity of Hg^2+^ toward other metals, which possibly coordinated through the surface carboxylate groups (Figure 5) of CDLP-D or through the byproducts formed. Selectivity toward carboxylate groups has also been observed in other mercury sensors^50,56−58^ and confirmed by FTIR and^1^ H NMR by Lin et al.^57^ Additionally, mercury may be adsorbed at CDLP-D binding sites, inducing selectivity for Hg^2+^ over other metals.^59,60^ Furthermore, as noted by Lin et al.,^57^ the presence of Hg^2+^ ions can promote π–π stacking interactions alongside carboxylate coordination, which likely contributes to the observed PL emission quenching.

The modified Stern–Volmer, eq 2, considers fractional accessibility of the fluorophores by the quenchers (Figure 4b), where *f*_a_ is the fraction of fluorescence available of the fluorophore and *K*_a_ the Stern–Volmer quenching constant of the accessible fraction.^10,39,52^ Based on the downward curvature in the Stern–Volmer plot and the aforementioned PL emission origin of CDLP-D, it is reasonable to apply this model to the studied system.
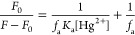
2

This model fitted better compared to the Stern–Volmer plot according to the coefficient of determination (*R*^2^) values in Table 1. This result suggests that the quenching mechanism between carbon dots and heavy metals must consider this model, as in most samples, PL emission originates from multiple populations rather than a single one. Ba et al.^39^ and Algarra et al.^61^ have also applied this model to carbon dot systems, with the latter study finding that 44% of fluorophores were accessible to Ag^+^ ions. Since in our sample surface carboxylate contributes to PL emission, Hg^2+^ quenching primarily affects these accessible states. Based on *f*_a_ values, this corresponds to approximately 35% of the total fluorophore emission being accessible. These findings indicate that certain regions of CDLP-D are inaccessible for Hg^2+^ quenching, which could explain the plateau observed in the sensing assay (Figure 3c). Even at a high Hg^2+^ concentration, the emission intensity was not completely quenched.

The difference between *K*_sv_ and *K*_a_, also verified by Ba et al.,^39^ possibly arises from the fact that the modified Stern–Volmer method assumes the static quenching mechanism, while the traditional Stern–Volmer model does not differentiate between dynamic and static quenching. This distinction underscores the importance of temperature assays in understanding quenching behavior. However, it is not totally clear why the *K*_a_ value increased from 288 to 298 K.

### 3.4 Detection of Hg^2+^ in a Real Water Sample

To investigate the application of Hg^2+^ detection by CDLP-D, tap water from the lab was used without pretreatment and spiking Hg^2+^, similar to Watcharamongkol et al.^30^ and Hama Aziz et al.^51^ After the spiked sample was incubated with the sensor, the PL emission was recorded. According to Table 2, the recoveries found were 97.6% and 108.5% (using eq 3) with a relative standard deviation (RSD, *n* = 3) of 5.1% and 0.9%, respectively. The results indicate a potential application of CDLP-D for Hg^2+^ sensing in real water samples.
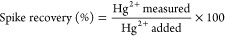
3

**Table 2:** Determination of Hg^2+^ in Tap Water by CDLP-D

added (μM)	measured ± SD (μM)	spike recovery (%)	% RSD (*n* = 3)
10.0	9.8 ± 0.5	97.6	5.1
30.0	32.6 ± 0.3	108.5	0.9

## 4 Conclusions

This paper addresses the need for monitoring heavy metals in water bodies due to their harmful effects on human health and the environment. Specifically, it demonstrates the application of carbon dot-LAPONITE nanocomposites (CDLP-D) for selective detection of mercury ions through the PL quenching effect. The produced CDLP-D sensors offer a rapid and efficient alternative due to their high quantum yield. In addition, the CDLP-D exhibits high photostability and sensitivity to Hg^2+^, with minimal interference from other metal ions, particularly Fe^3+^, which is the most common interference with Hg^2+^ sensors, in the optimal concentration range of 1–40 μM. The quenching mechanism was explored using Stern–Volmer models, revealing that static quenching is predominant at low Hg^2+^ concentrations, while collisional quenching occurs at higher concentrations.

Additionally, studies on the quenching mechanism between carbon dots and heavy metals must consider the fractional accessibility model, as the PL emission of carbon dots is not fully accessible to quenchers. In our case, only 35% of the emission was accessible to Hg^2+^, suggesting opportunities for the further optimization of surface functional groups to enhance Hg^2+^ detection. While the CDLP-D sensor shows promising potential for Hg^2+^ detection, in situ environmental analysis presents challenges due to variables such as pH fluctuations, temperature changes, and the presence of organic matter in natural water bodies, which may impact sensor performance. Future studies should investigate these real-world conditions and explore the integration of this sensor with optical fiber technology to enable practical, on-site monitoring applications.

## Supporting Information Available

Emission spectra of CD as a function of pH and concentration; relative emission integrated area of CDLP-D and CD using 100 μM; PL emission spectra of CD as a function of Fe^3+^ and Hg^2+^ concentrations from 0 to 900 μM; emission decay at room temperature (298 K) for CDLP-D samples in water suspension; and carbon dots-based PL sensor for Hg^2+^ detection (PDF)
