# Supplementary material for: Carbon Dot-Laponite Hybrid Nanocomposites as Selective Turn-Off Sensors for Hg2+ Detection and Photoluminescence Quenching Mechanism
Source: ACS Omega. 2024 Dec 17;9(52):51204–12. doi: 10.1021/acsomega.4c07183 (PMC11696425; doi:10.1021/acsomega.4c07183)
Supplement: Supplementary file 1 — ao4c07183_si_001.pdf [file ao4c07183_si_001.pdf]

Carbon Dot-Laponite Hybrid Nanocomposites as Selective Turn-Off Sensors for Hg^{2+} Detection and Photoluminescence Quenching Mechanism

Bruno S. D. Onishi,[a,]* Albano N. Carneiro Neto,[b] Sidney J. L. Ribeiro[a,]*

[a] Institute of Chemistry, São Paulo State University – UNESP, São Paulo, Araraquara, 14800-060, Brazil.

[b] Physics Department and CICECO – Aveiro Institute of Materials, University of Aveiro, 3810-193 Aveiro, Portugal.

* Corresponding authors email: bruno.onishi@unesp.br; sidney.jl.ribeiro@unesp.br

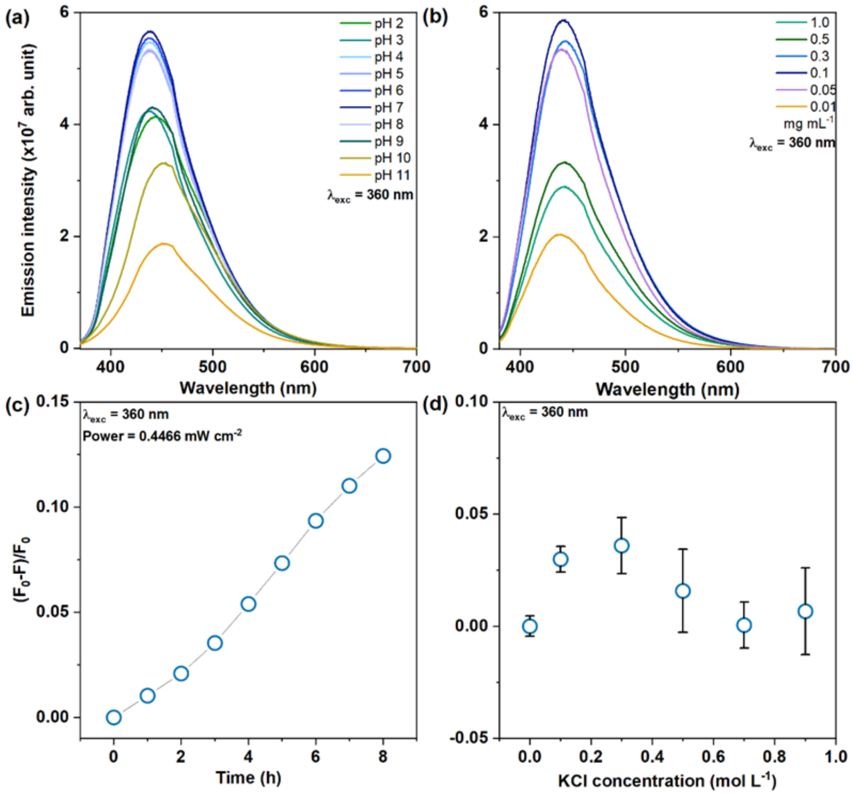

Figure S1. Emission spectra of CD as a function of (a) pH and (b) concentration; relative integrated area of emission band as a function of (c) time and (d) ionic strength – F_0 is the integrated area of the CD emission spectrum at (c) 0h and (d) 0 mol·L^{-1} KCl, and F is the integrated area of the CD emission spectrum at (c) x (x = 1 to 8) hours and (d) y (y = 0.1 to 0.9) mol L^{-1}. For (a), (c), and (d) the CD concentration was maintained at 0.1 mg·mL^{-1}.

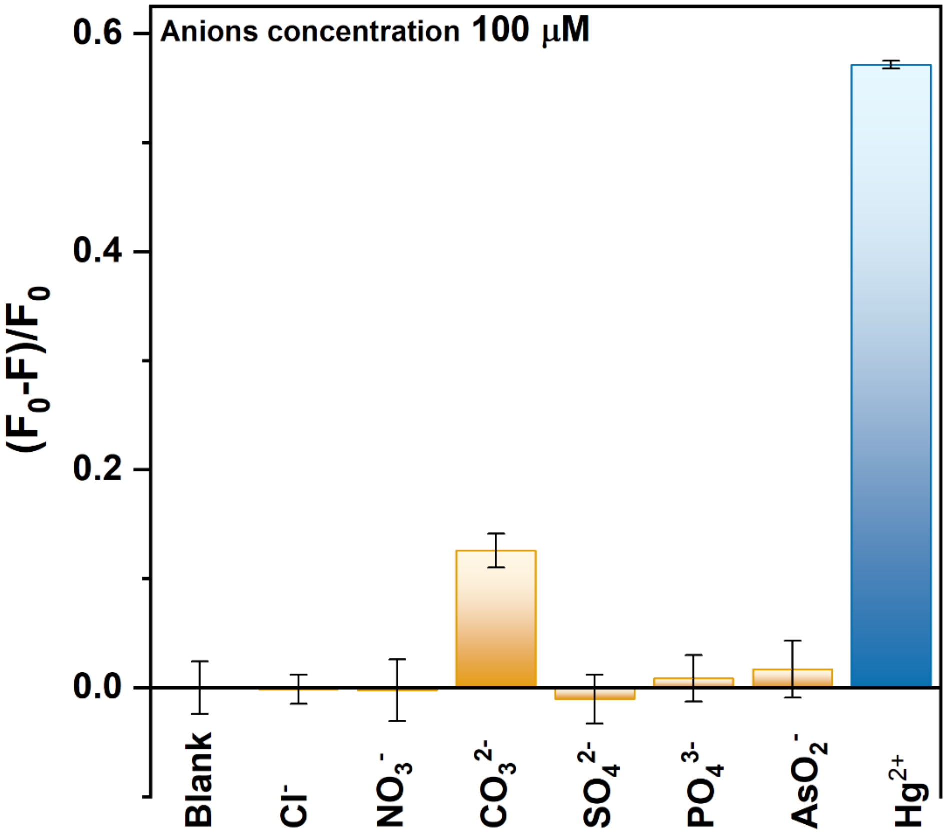

Figure S2. Relative emission integrated area of CDLP-D using 100 μM of as Cl^-, NO_3^-, CO_3^{2-}, SO_4^{2-}, PO_4^{3-} and AsO_2 (yellow bars) and Hg^{2+} (blue bar). F_0 is the blank integrated area emission (CDLP-D) and F is the integrated area emission in the presence of the respective ions.

Table S1 – Table of carbon dots-based PL sensor for Hg^{2+} detection, whereas LOD is the Limit of Detection and the references are provided in the main text.

**Carbon source**	**Synthesis condition**	**Linear range**	**LOD**	**Reference**
Citric Acid and 2-aminobenzimidazole	Solid-state method using autoclave at 180 ºC for 5h	12-28 μM	3.5 μM	29
Cassava pulp	Hydrothermal at 200 ºC for 12h	30 – 600 μM	12 μM	30
Citri acid, urea and L-cysteine	Microwave at 800 W for 2 to 10 min	0-40 μM	2 μM	31
**Citric acid, ethylenediamine and Laponite**	**Hydrothermal at 180 ºC for 6h**	**1-40 μM**	**2.5 μM**	**This work**
Pigeon feathers	Pyrolysis at 300 ºC for 3h	0.01-0.12 μM	10.3 nM	50
Aspartic acid, D-glucose and polyethylene imine branched	Hydrothermal at 180 ºC for 20h	0.02 – 1 μM	10 nM	51

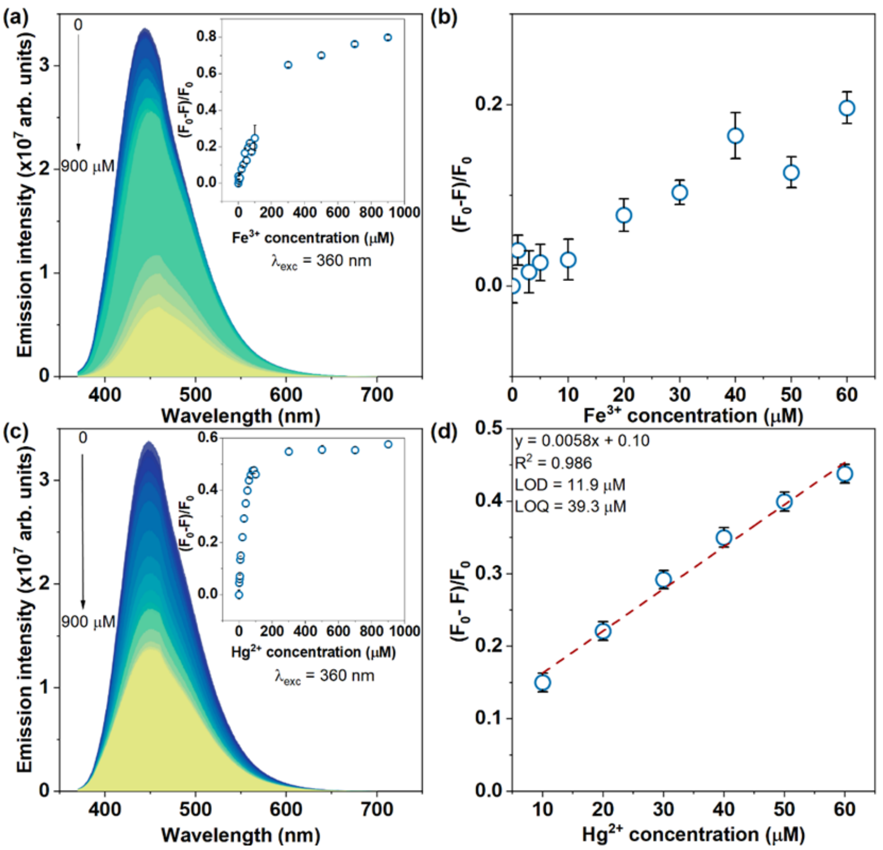

Figure S3. PL emission spectra of CD as a function of (a) Fe^{3+} and (b) Hg^{2+} concentration from 0 to 900 μM. The insert graph in (a) and (c) are the relative integrated emission area as a function of Fe^{3+} and Hg^{2+} concentrations in the 0-900 μM range, respectively, as well as (b) and (d) in the 0-40 μM range.

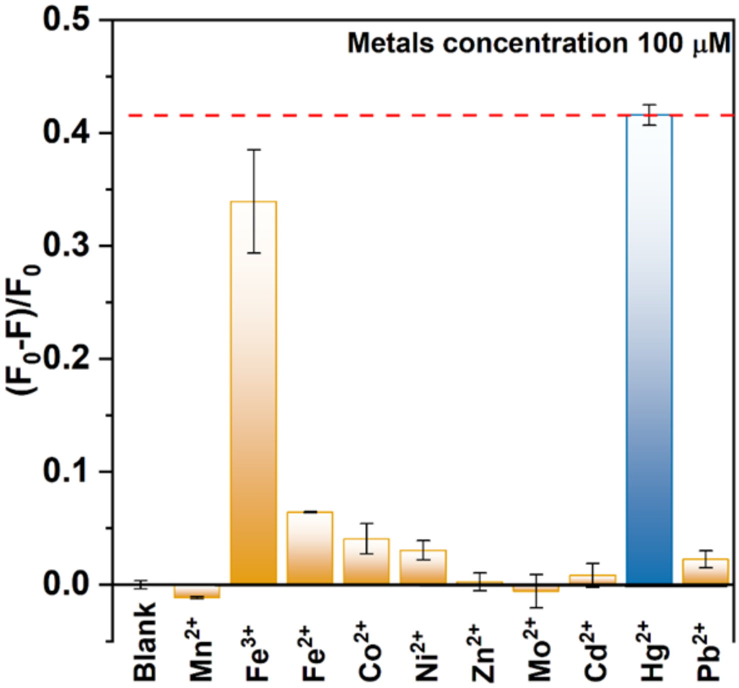

Figure S4. Relative emission integrated area of CD using 100 μM of Mn^{2+}, Fe^{3+}, Fe^{2+}, Co^{2+}, Ni^{2+}, Zn^{2+}, Mo^{2+}, Cd^{2+}, Pb^{2+} (yellow bars) and with Hg^{2+} (blue bar). F_0 is the blank integrated area emission (CD), while F is the integrated area emission in the presence of the respective metals.

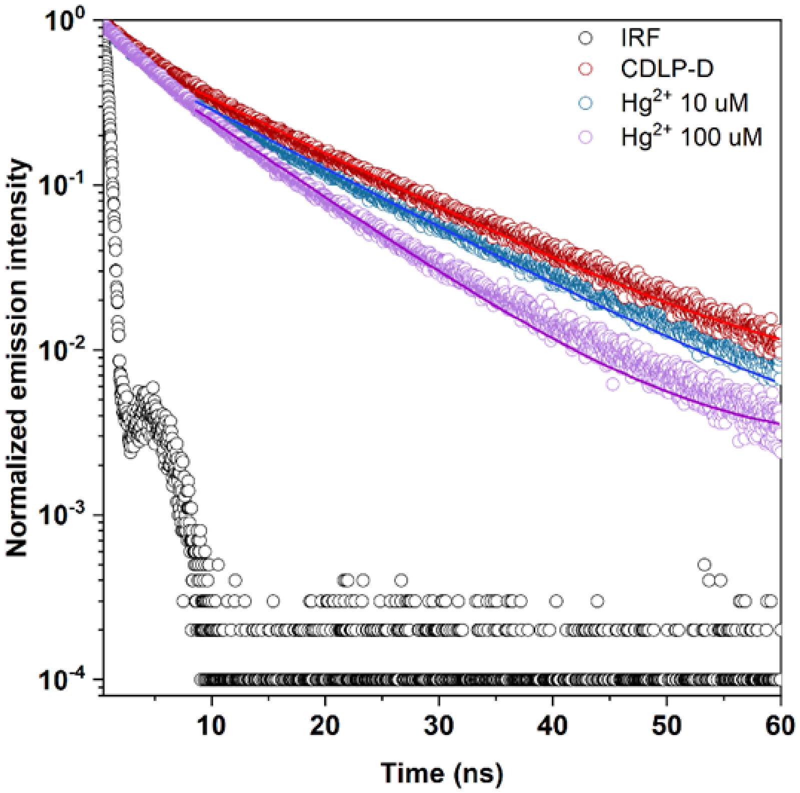

Figure S5. Emission decay at room temperature (298 K) for CDLP-D samples in water suspension (red), and in the presence of Hg^{2+} 10 μM (blue) and 10 μM (violet) using a 350 nm nanoLED for excitation and monitoring emission at 450 nm. IRF is the instrumental response function. Lifetime (τ) was obtained by fitting emission decay (solid lines). The model used was $y = y_0 + \sum_n A_n exp\,(-(t_n - x_0)/\tau_n)$, where y is the PL emission intensity, y_0 is a constant, A_n is the amplitude of the n component of the decay, t_n is the experimental decay data, and x_0 = 8.7 ns is obtained by the IRF. The best fitting was achieved by applying one component.
